# Can vaccinia virus be replaced by MVA virus for testing virucidal activity of chemical disinfectants?

**DOI:** 10.1186/1471-2334-10-185

**Published:** 2010-06-23

**Authors:** Holger F Rabenau, Ingrid Rapp, Jochen Steinmann

**Affiliations:** 1 Institute of Medical Virology, Hospital of the Johann Wolfgang Goethe University of Frankfurt, Paul-Ehrlich-Str. 40, 60596 Frankfurt am Main, Germany; 2 Labor Dr. Merk & Kollegen, Beim Braunland 1, 88416 Ochsenhausen, Germany; 3 MikroLab GmbH, Norderoog 2, 28259 Bremen, Germany

## Abstract

**Background:**

Vaccinia virus strain Lister Elstree (VACV) is a test virus in the DVV/RKI guidelines as representative of the stable enveloped viruses. Since the potential risk of laboratory-acquired infections with VACV persists and since the adverse effects of vaccination with VACV are described, the replacement of VACV by the modified vaccinia Ankara strain (MVA) was studied by testing the activity of different chemical biocides in three German laboratories.

**Methods:**

The inactivating properties of different chemical biocides (peracetic acid, aldehydes and alcohols) were tested in a quantitative suspension test according to the DVV/RKI guideline. All tests were performed with a protein load of 10% fetal calf serum with both viruses in parallel using different concentrations and contact times. Residual virus was determined by endpoint dilution method.

**Results:**

The chemical biocides exhibited similar virucidal activity against VACV and MVA. In three cases intra-laboratory differences were determined between VACV and MVA - 40% (v/v) ethanol and 30% (v/v) isopropanol are more active against MVA, whereas MVA seems more stable than VACV when testing with 0.05% glutardialdehyde. Test accuracy across the three participating laboratories was high. Remarkably inter-laboratory differences in the reduction factor were only observed in two cases.

**Conclusions:**

Our data provide valuable information for the replacement of VACV by MVA for testing chemical biocides and disinfectants. Because MVA does not replicate in humans this would eliminate the potential risk of inadvertent inoculation with vaccinia virus and disease in non-vaccinated laboratory workers.

## Background

The global eradication of variola virus is one of the greatest achievements of modern medicine. Following this eradication, routine childhood vaccination against the causative agent was halted because of a declining probability of the importation and spread and occasional serious side effects of the vaccination [1]. In Europe the vaccinia virus strain Lister Elstree (VACV) was used [2]. Thereafter, for nearly three decades (1972 to 2003), American laboratory workers were the only group put forward for periodic smallpox vaccination. The Advisory Committee on Immunization Practices (ACIP) recommend a smallpox booster vaccination at least every 10 years for those employees who handle non-highly attenuated vaccinia virus or other orthopoxviruses (e.g., monkeypox) [3].

Due to the incidence of serious side effects in immunocompetent individuals (about 1/500,000) a more attenuated vaccinia virus with a high safety profile for mammalian species was developed - the so-called modified vaccinia Ankara strain (MVA) [4,5]. In the meantime MVA is a widely used tool in molecular biology in laboratories around the world.

Presently the vaccinia virus Lister Elstree is still used as model virus for testing the virucidal activity of chemical disinfectants. In Europe the norm EN 14476:2007 exists for determining virucidal activity [6]. This norm is only designed to determine "complete virucidal activity" (i.e. enveloped and non-enveloped viruses) which is not needed in all cases, e.g. for inactivation of blood-borne viruses. The number of active substances achieving virucidal activity is limited. Taking into consideration compatibility and environmental implications, a German national guideline [7] for human medicine permits additional tests where a disinfectant only has "limited virucidal activity" (active against enveloped viruses such as HIV, HBV, and HCV) [8], which means that it has proven activity against two representatives of enveloped viruses - vaccinia virus and bovine viral diarrhea virus (BVDV). Apart from this aspect, the consequence of the guideline is that despite a growing number of humans without immunity against vaccinia virus, VACV is still used in laboratories to test the virucidal efficacy of chemical disinfectants. As model viruses for "non-enveloped" viruses the guidelines of the German Association for the Control of Virus Diseases (DVV) and the Robert Koch-Institute (RKI) [7] prescribes poliovirus type 1 vaccine strain LSc-2ab, adenovirus type 5 strain Adenoid 75 and polyomavirus SV 40 strain 777.

In veterinary medicine vaccinia virus strain Lister Elstree, ECBO-virus strain LCR-4, reovirus type 1 and Newcastle-Disease-Virus strain Montana are required for virucidal testing of chemical disinfectants, pursuant to the guideline of the German Veterinary Medical Society (DVG) [9].

The DVV/RKI guidelines state that laboratory workers handling vaccinia virus should be vaccinated [7]. However, there are concerns about the adverse effects of vaccination with VACV. This objection has arisen as a result of vaccinations against smallpox which have been performed as a consequence of and as preventive measure against putative bioterrorist attacks [10-12].

In the USA it is not necessary to report laboratory-acquired VACV infections - nevertheless between 2005 and 2007, five cases were reported to the CDC [13]. These infections typically occurred in unvaccinated workers [14] and could be associated with high inoculum and inoculation routes with a high risk of complications [15]. Additionally, in other parts of the world several reports exist regarding inadvertent inoculation and laboratory-acquired VACV infections [16-21].

Within this context it is important to highlight that in a recently published study comparable results could be shown when using MVA instead of vaccinia virus strain Lister Elstree when testing chemical disinfectants for virucidal activity. This study was conducted according to the DVG guidelines with quantitative suspension and qualitative carrier tests [21].

In order to investigate the possibility of substituting VACV with MVA under the guidelines of the DVV/RKI, different biocides (peracetic acid, aldehydes and alcohols), which are often ingredients used in disinfectants, were tested with quantitative suspension tests using VACV and MVA in parallel. The tests were performed in the presence of organic load. Furthermore, the aim of the study was to evaluate the robustness of the testing method when performing an inter-laboratory comparison (as an external quality assurance programme (EQA)). Therefore, the results of three laboratories were compared using statistical analysis as required by the DVV/RKI guidelines [7].

## Methods

### Participants in the external quality assurance program (EQA)

Three German laboratories participated in this EQA program (a list of participants is given in the acknowledgements section) - lab. 1, lab 2, lab. 3, respectively. Participation was open and free of charge to all laboratories.

### Viruses and cell cultures

MVA (kindly provided by Prof. Dr. Truyen, Leipzig) were propagated in BHK-21 (Baby hamster kidney cells, kindly provided by Friedrich Löffler Institut, Insel Riems, Germany) and VACV (ATCC VR-1549) in African green monkey kidney cells (Vero, ATCC CRL-1586). For virus propagation cells were grown at 37°C and 5% CO_2 _in minimum essential medium (MEM) supplemented with 10% foetal calf serum (FCS). These cells were infected with a multiplicity of infection of 0.1. After cells exhibited a cytopathic effect, they were subjected to a twofold freeze/thaw procedure followed by a low speed centrifugation (10 min and 1000 × g) in order to sediment cell debris. After aliquoting, test virus suspension was stored at -80°C.

### Biocides

Five biocides were used in the study: formaldehyde (0.7%, w/v; pH 7; contact time: 5, 15, 30, 60 min), glutardialdehyde (0.05, 0.1, 0.5%, w/w; pH 8.4; contact time: 0.5, 2, 5 min), ethanol (30, 40, 50, 60%, v/v; contact time: 1, 2 min), isopropanol (20, 30, 40, 50, 60%, v/v; contact period: 1, 2 min), and peracetic acid (PAA) (0.001, 0.0025, 0.005, 0.01, 0.05, 0.1%, v/v; contact time: 1, 2 min). The pH values of the biocides were measured if necessary.

### Preparation of test samples

Test samples for the proficiency panel (stock solutions) were generated by an independent provider (kindly provided by Dr. von Rheinbaben, Ecolab Deutschland GmbH, Düsseldorf, Germany). All samples were sent out by a commercial parcel service and arrived within 24 h of dispatch, according to the date of receipt provided by the participants. The participants were asked to analyze the material according to the method described in the German DVV/RKI guidelines [7].

### Determination of cytotoxicity

In order to determine cytotoxicity biocides were serially diluted tenfold in MEM up to a dilution of 10^-8^. One part by volume of water of standardized hardness (instead of virus suspension) was mixed with one part by volume of organic load and eight parts by volume of the biocide. Aliquots of 50-100 μl from each test concentration and each dilution was then inoculated into seven to eight wells of a 96-well microtitre plate containing 50 μl cell suspension of Vero or BHK-21 cells, respectively. The cell cultures were observed for cytotoxic effects for the same incubation time as afterwards used for the suspension tests.

### Quantitative suspension tests

Tests were carried out in accordance with the DVV/RKI guidelines at 20°C [7]. One part by volume of virus suspension (titre of at least 10^7^-10^8 ^tissue culture infectious dosis 50% (TCID_50_)/ml) and one part by volume of the organic load were mixed with eight parts by volume of the biocide. The tests were carried out in ambient temperatures of 20-22°C. Infectivity was determined by means of end point dilution titration in microtitre plates. At the end of the chosen exposure time, activity of the biocides was immediately stopped by serial dilutions with ice-cold cell culture medium. 50 μl from each dilution were placed in seven to eight wells of a sterile polystyrene flat-bottomed 96-well microtitre plate containing 50 μl cell suspension (10-15 × 10^3 ^cells per well) of Vero or BHK-21 cells. Cultures were observed for cytopathic effects (CPE) after seven to ten days of inoculation. All tests were conducted in two independent tests run on different days. Virus controls were incorporated after the longest exposure time. All tests were performed with an organic load of 10% FCS.

### Statistical analysis

The virus titres were determined using the method of Spearman and Kaerber [22,23] and expressed as TCID_50_/ml including standard deviation. Titre reduction is presented as the difference between the virus titre after contact time with the substance and control virus titre. This difference is given as reduction factor (RF) including its 95% confidence interval (CI). A reduction of infectivity of ≥4 log_10 _steps (inactivation ≥99.99%) was regarded as evidence of sufficient virucidal activity. The calculation was performed according to the DVV/RKI guidelines [7]. Biologically relevant RF differences between viruses or laboratories were defined as ≥1 log_10 _step considering the lower respectively upper bounds of the 95% CI.

## Results

**Figure 1:**
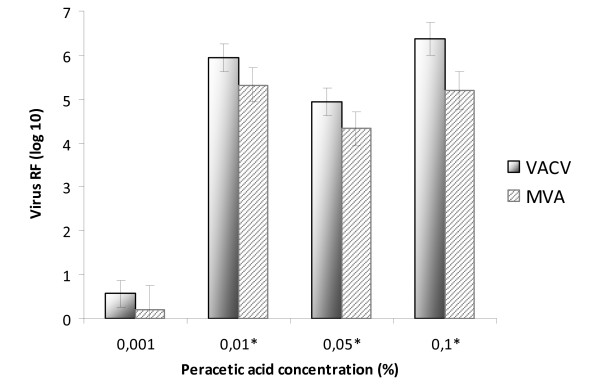
**Concentration-dependent virucidal activity of peracetic acid against VACV and MVA (shown are exemplary values for one of the test laboratories)**. *At this concentration the measurable RF was limited by cytotoxic reactions and/or the amount of infectious viruses in the virus stock).

The test concentrations and contact periods were chosen in order to observe a kinetic and the transition from non-efficient to efficient virus inactivation. For ethanol a concentration-dependent virucidal activity was seen for VACV as well as for MVA. At concentrations ≥50% (v/v) a maximum titre reduction of at least 10^3.6 ^TCID_50_/ml was observed after 1 min with both - VACV (Table 1) and MVA (Table 2). Testing a concentration of 40% (v/v) in all three laboratories residual infectivity could be detected after one or two minutes. A complete inactivation of VACV and MVA was also detected for isopropanol and PAA at concentrations of ≥40% (v/v) and 0.01%, respectively (Table 1, 2). At lower concentration levels intra-laboratory heterogeneous discrepancies between VACV and MVA could be seen (Figure 1). Furthermore, in some cases inter-laboratory discrepancies were obvious for the same virus.

**Table 1:** Virucidal activity of ethanol, isopropanol and peracetic acid (PAA) against VACV depending on concentration and contact time

	VACV reduction factor (RF) (log_10_)		
**biocide**	**results lab 1**	**results lab 2**	**results lab 3**

	**1 min**	**1 min**	**1 min**

30% ethanol	0.13 ± 0.38	-0.25 ± 0.33	≥0.14 ± 0.60

40% ethanol	2.26 ± 0.36	2.69 ± 0.54	3.86 ± 0.48

50% ethanol	≥4.38 ± 0.37	≥5.94 ± 0.31	≥5.0 ± 0.40

60% ethanol	≥4.38 ± 0.37	≥5.94 ± 0.31	≥5.0 ± 0.40

20% isopropanol	0.82 ± 0.27	0.00 ± 0.52	-0.57 ± 0.45

30% isopropanol	0.32 ± 0.34	1.87 ± 0.25	1.86 ± 0.48

40% isopropanol	≥4.38 ± 0.37	≥5.94 ± 0.31	≥5.0 ± 0.40

50% isopropanol	≥4.38 ± 0.37	≥5.94 ± 0.31	≥5.0 ± 0.40

60% isopropanol	≥4.38 ± 0.37	≥5.94 ± 0.31	≥5.0 ± 0.40

0.001% PAA	0.94 ± 0.41	0.56 ± 0.48	0.71 ± 0.53

0.0025% PAA	3.38 ± 0.52	n.d.	n.d.

0.005% PAA	≥4.50 ± 0.38	n.d.	n.d.

0.01% PAA	≥3.50 ± 0.38	≥5.94 ± 0.31	≥4.0 ± 0.40

0.05% PAA	≥3.50 ± 0.38	≥4.94 ± 0.31	≥4.0 ± 0.40

0.1% PAA	≥4.57 ± 0.41	≥6.38 ± 0.37	≥3.86 ± 0.39

The prefix "≥" indicates that the determination of the RF is limited by cytotoxic reactions and/or the amount of infectious viruses in the test virus suspension

n.d. = not done

**Table 2:** Virucidal activity of ethanol, isopropanol and peracetic acid (PAA) against MVA depending on concentration and contact time

	MVA reduction factor (RF) (log_10_)		
**biocide**	**results lab 1**	**results lab 2**	**results lab 3**

	**1 min**	**1 min**	**1 min**

30% ethanol	-0.10 ± 0.37	-0.06 ± 0.51	-0.14 ± 0.62

40% ethanol	2.80 ± 0. 53	≥5.32 ± 0.38	1.71 ± 0.60

50% ethanol	≥5.40 ± 0.36	≥5.32 ± 0.38	≥3.57 ± 0.40

60% ethanol	≥5.40 ± 0.36	≥5.32 ± 0.38	≥3.57 ± 0.40

20% isopropanol	0.06 ± 0.37	-0.44 ± 0.63	0.42 ± 0.46

30% isopropanol	0.50 ± 0.44	3.69 ± 0.53	0.71 ± 0.60

40% isopropanol	≥5.40 ± 0.36	≥5.32 ± 0.38	≥3.57 ± 0.40

50% isopropanol	≥5.40 ± 0.36	≥5.32 ± 0.38	≥3.57 ± 0.40

60% isopropanol	≥5.40 ± 0.36	≥5.32 ± 0.38	≥3.57 ± 0.40

0.001% PAA	0.90 ± 0.47	0.19 ± 0.56	1.14 ± 0.56

0.0025% PAA	4.75 ± 0.31	n.d.	n.d.

0.005% PAA	≥5.66 ± 0.47	n.d.	n.d.

0.01% PAA	≥5.66 ± 0.47	≥5.32 ± 0.38	≥3.57 ± 0.56

0.05% PAA	≥4.60 ± 0.47	≥4.32 ± 0.38	≥4.14 ± 0.40

0.1% PAA	≥3.94 ± 0.47	≥5.19 ± 0.43	≥4.14 ± 0.40

The prefix "≥" indicates that the determination of the RF is limited by cytotoxic reactions and/or the amount of infectious viruses in the test virus suspension

n.d. = not done

**Figure 2:**
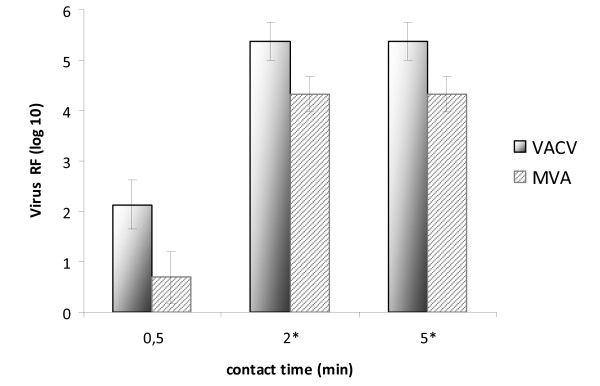
**Time-dependent virucidal activity of 0.1% glutardialdehyde against VACV and MVA (shown are exemplary values for one of the test laboratories)**. *After this contact time the measurable RF was limited by cytotoxic reactions and/or the amount of infectious viruses in the virus stock).

**Figure 3:**
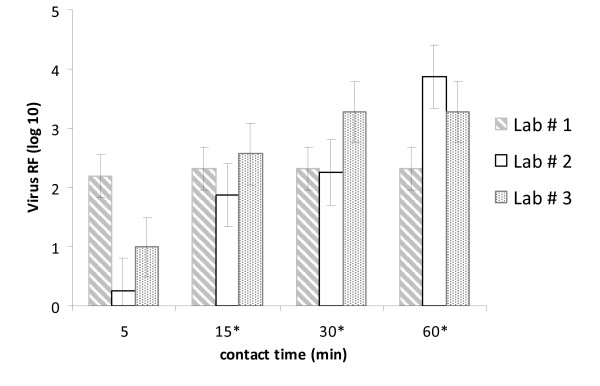
**Time-dependent virucidal activity of 0.7% formaldehyde against VACV tested in all 3 laboratories**. *After this contact time the measurable RF in at least one of the laboratories was limited by cytotoxic reactions and/or the amount of infectious viruses in the virus stock.

A time-dependent increase of virucidal activity of the biocides was detected when testing lower concentrations (not shown in table). Concerning glutardialdehyde a time - and concentration-dependent increase of virucidal activity were detectable for the 0.1% and 0.05% concentrations for both viruses (Table 3, Figure 2). In contrast, for formaldehyde a time-dependent RF increase could be seen only for VACV (Figure 3), whilst the maximal measurable RF for MVA was already apparent after the shortest incubation period of 5 min (data not shown).

**Table 3:** Virucidal activity of glutardialdehyde against VACV and MVA virus depending on concentration and contact time

	VACV RF (log_10_)								
**Biocide**	**results lab 1**			**results lab 2**			**results lab 3**		

	**0.5 min**	**2 min**	**5 min**	**0.5 min**	**2 min**	**5 min**	**0.5 min**	**2 min**	**5 min**

0.05% glutardialdehyde	0.50 ± 0.34	4.06 ± 0.32	≥4.82 ± 0.29	0.50 ± 0.44	3.88 ± 0.53	≥5.38 ± 0.37	≥3.86 ± 0.49	≥4.43 ± 0.49	≥4.58 ± 0.49

0.1% glutardialdehyde	2.75 ± 0.39	≥3.82 ± 0.29	≥3.82 ± 0.29	2.13 ± 0.49	≥5.38 ± 0.37	≥5.38 ± 0.37	≥3.86 ± 0.39	≥3.86 ± 0.39	≥3.86 ± 0.39

0.5% glutardialdehyde	≥2.82 ± 0.29	≥2.82 ± 0.29	≥2.82 ± 0.29	≥5.38 ± 0.37	≥5.38 ± 0.37	≥5.38 ± 0.37	≥4.0 ± 0.35	≥4.0 ± 0.35	≥4.0 ± 0.35

	**MVA RF (log_10_)**								

**Biocide**	**results lab 1**			**results lab 2**			**results lab 3**		

	**0.5 min**	**2 min**	**5 min**	**0.5 min**	**2 min**	**5 min**	**0.5 min**	**2 min**	**5 min**

0.05% glutardialdehyde	1.38 ± 0.41	≥3.88 ± 0.42	≥4.50 ± 0.38	0.19 ± 0.56	2.07 ± 0.48	≥4.19 ± 0.43	1.86 ± 0.53	≥3.14 ± 0.40	≥3.14 ± 0.40

0.1% glutardialdehyde	2.81 ± 0.38	≥4.00 ± 0.38	≥4.00 ± 0.38	0.69 ± 0.51	≥4.32 ± 0.35	≥4.32 ± 0.35	≥3.14 ± 0.40	≥3.14 ± 0.40	≥3.14 ± 0.40

0.5% glutardialdehyde	≥3.50 ± 0.38	≥3.50 ± 0.38	≥3.50 ± 0.38	≥3.32 ± 0.35	≥3.32 ± 0.35	≥3.32 ± 0.35	≥3.14 ± 0.40	≥3.14 ± 0.40	≥3.14 ± 0.40

The prefix "≥" indicates that the determination of the RF is limited by cytotoxic reactions and/or the amount

The standard deviations for the control virus titrations were below 0.5 log_10 _TCID_50_/ml (not shown in tables).

In many cases the RF is marked with the prefix "≥" indicating that the determination of the RF was limited by cytotoxic reactions and/or the amount of infectious viruses in the virus stock. Furthermore, the different levels on test virus suspension are to some extent responsible for inter-laboratory and inter-virus RF differences.

**Figure 4:**
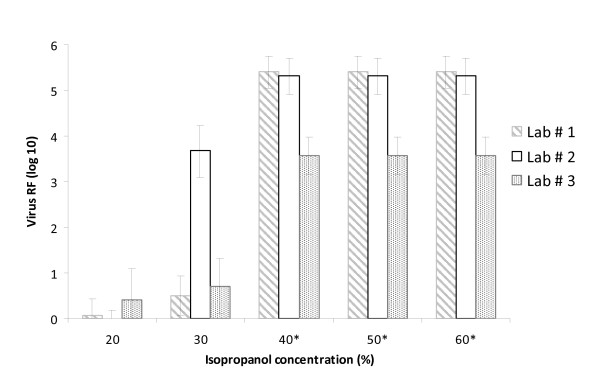
**Concentration-dependent virucidal activity of isopropanol against MVA tested in all 3 laboratories (the minor absolute RF of Lab 3 is related to the lower starting virus titre)**. *At this concentration the measurable RF was limited by cytotoxic reactions and/or the amount of infectious viruses in the virus stock.

For the different biocides all RF data were compared with regard to relevant intra- and inter-laboratory differences of viable VACV and MVA. In two cases remarkable inter-laboratory RF differences (≥1 log_10 _step considering 95% CI) were seen. The different results concerned in both cases MVA (for 40% ethanol and 30% isopropanol) (Figure 4). Furthermore, in three cases intra-laboratory differences were demonstrated between VACV and MVA (for 40% ethanol, 30% isopropanol, 0.05% glutardialdehyde) (Tables 1, 2, 3).

## Discussion

VACV is still used in many laboratories - e.g. as a model virus for a large enveloped DNA virus. The virus is also required in the German guidelines of DVV/RKI and DVG as a model virus for testing the virucidal efficacy of chemical disinfectants [7,9].

Before eradication of variola the risk of human laboratory infection with VACV was negligible because most of the population was vaccinated with VACV [24]. As a long-term consequence of the smallpox eradication the number of people with a sufficient immunity has decreased. This trend is higher in younger laboratory workers where no orthopox basic immunity exists and the potential risk of laboratory-acquired infections persists [13,14,16-20].

To diminish this risk one possibility would be to vaccinate these laboratory workers, but there are concerns about the adverse effects of vaccination with VACV because such side effects are about 10 times more common in primary vaccinees than in those who are revaccinated [25]. Therefore, it would be worthwhile to substitute VACV with MVA.

The present study was undertaken with two aims in mind: first to evaluate a possible substitution of VACV with MVA and second to investigate the robustness of the test in evaluating the laboratory proficiency in a multi-center study. Three different laboratories participated in this EQA.

By testing five chemical biocides from different groups (peracetic acid, aldehydes, and alcohols) in a quantitative suspension test [7] we were able to show that they exhibited similar virucidal activity against VACV and MVA or - put differently - that both viruses revealed comparable stability and susceptibility to the tested biocides. This data confirms the results from Hartnack et al. [21], who achieved similar results testing four different DVG-listed commercially available chemical disinfectants representing different groups of chemicals in quantitative suspension tests and qualitative carrier tests with poplar wood and gauze. The high and very similar viability of VACV and MVA had been previously confirmed in different studies [26-29].

In our study we chose the concentration of the biocides and the length of the exposure time in order to see a kinetic and the transition from non-efficient to efficient virus inactivation. For the different biocides all RF data were compared with regard to relevant (≥1 log_10 _step considering 95% CI) intra- and inter-laboratory differences of viable VACV and MVA. Marked inter-laboratory RF differences were only observed in two cases; in three cases intra-laboratory differences between VACV and MVA were demonstrated.

Overall MVA appears to have a similar stability as VACV, but in some cases it seems to be more fragile (e.g. at 40% ethanol and 30% isopropanol). In contrast, when testing with 0.05% glutardialdehyde, MVA seems to be more stable than VACV. One reason for these differences could be that in the selection of concentration and contact time-relation the kinetic was in the ascending proportion of the sigmoid area of the inactivation curve. These conditions were chosen in order to see a kinetic and the transition from non-efficient to efficient virus inactivation. On the other hand this would mean that even the slightest change in conditions or the test procedure could cause a maximum variance and lead to measurable changes in the results. Therefore, the RF variations do not appear to be attributable to relevant differences in the tenacity of both viruses. Furthermore, differences in the tenacity of both viruses are only found at concentrations far below the standards applied in practice.

This is the first multi-center German EQA study on testing the virucidal activity of chemical biocides according to the DVV/RKI guidelines. Comparative testing of defined samples is the most efficient method to identify weaknesses in a laboratory or in certain methodological components. Our data shows that the test accuracy across the three participating laboratories is high. In all cases the standard deviations for the control virus titrations were below 0.5 log_10 _TCID_50_/ml. Observed RF discrepancies are found at a concentration of low activity and might be caused by different preparation of the virus test suspension, usage of different cell lines (Vero or BHK-21 cells), limitations by cytotoxic reactions and the aforementioned strong influence of experimental conditions due to the inactivation kinetic.

## Conclusion

As stated by Hartnack et al. [21] our data confirms and underlines that MVA could substitute VACV in the chemical disinfectant testing guidelines [7,9]. Because MVA does not replicate in humans this would eliminate the potential risk of inadvertent inoculation with vaccinia virus and disease in non-vaccinated laboratory workers.

## Competing interests

The authors declare that they have no competing interests.

## Authors' contributions

HR: author of the publication, also provided analysis and interpretation of data, responsible for study design. IR and JS: Co-authors of the publication and responsible for study design, also contributed analysis and interpretation of data. All authors have read and approved the final manuscript

## Pre-publication history

The pre-publication history for this paper can be accessed here:

http://www.biomedcentral.com/1471-2334/10/185/prepub
